# Surgical Management of Pericardial Effusion of Non-cardiac Etiology: Etiologic Spectrum, Diagnostic Contribution, and Early Outcomes in a Single-Center Retrospective Case Series

**DOI:** 10.7759/cureus.111612

**Published:** 2026-06-27

**Authors:** Rendy Agustian, Rini Istisakinah, Yudri Adrian, Sedayu, Dwi Agustina, Wahyudi, Hani Andriani, Syifa Nurlathifah

**Affiliations:** 1 Department of Surgery, Division of Cardiothoracic and Vascular Surgery, Waled Regional General Hospital, Cirebon, IDN; 2 Department of Surgery, Division of Cardiothoracic and Vascular Surgery, Swadaya Gunung Jati University, Cirebon, IDN; 3 Department of Internal Medicine, Division of Cardiology, Waled Regional General Hospital, Cirebon, IDN; 4 Department of Internal Medicine, Division of Cardiology, Swadaya Gunung Jati University, Cirebon, IDN; 5 Department of Internal Medicine, Waled Regional General Hospital, Cirebon, IDN; 6 Department of Internal Medicine, Swadaya Gunung Jati University, Cirebon, IDN; 7 Department of Internal Medicine, Division of Pulmonology, Waled Regional General Hospital, Cirebon, IDN; 8 Department of Internal Medicine, Division of Pulmonology, Swadaya Gunung Jati University, Cirebon, IDN; 9 Department of Pathological Anatomy, Waled Regional General Hospital, Cirebon, IDN; 10 Department of Pathological Anatomy, Swadaya Gunung Jati University, Cirebon, IDN

**Keywords:** cardiac tamponade, diagnostic contribution, pericardial effusion, pericardiectomy, pericardiotomy, tuberculous pericarditis, vast, video-assisted thoracoscopic surgery

## Abstract

Background

Pericardial effusion has many causes, ranging from inflammatory and infectious diseases to tuberculosis, malignancy, renal failure, autoimmune conditions, and systemic illness. Pericardiocentesis provides rapid relief of pericardial effusion. However, in certain non-cardiac etiologies, fluid analysis alone may be insufficient to establish the underlying diagnosis. Surgical drainage through pericardiotomy or pericardiectomy allows controlled evacuation and direct sampling of the pericardium, which can help establish the diagnosis and guide the treatment.

Objective

The main objective of this study is to describe the presentation, echocardiographic features, etiologic spectrum, surgical approaches, diagnostic contribution, and early outcomes of patients who underwent surgery for pericardial effusion at a tertiary referral center in a tuberculosis-endemic region.

Methods

We reviewed 15 consecutive adolescent and adult patients with moderate-to-large pericardial effusion treated surgically between January 2023 and December 2025. Demographics, presentation, echocardiographic findings, operative approach, microbiology, cytology, histopathology, length of stay, and in-hospital outcome were recorded and analyzed descriptively. We defined diagnostic contribution as any histopathologic, cytologic, microbiologic, or clinically integrated information from surgery that helped classify the etiology and the impact on management as a surgical finding that started, confirmed, or changed treatment.

Results

Median age was 45 years (interquartile range (IQR), 32-55; range, 15-83), and nine patients (60%) were women. Every patient was dyspneic, and 10 patients (66.7%) had cardiac tamponade or impending tamponade. The largest effusion dimension on echocardiography ranged from 16 to 50 mm. Six patients (40%) underwent subxiphoid pericardiotomy, eight (53.3%) underwent video-assisted thoracoscopic surgery (VATS) pericardiotomy, and one (6.7%) underwent VATS pericardiectomy. Surgical drainage was successfully completed in all patients and provided therapeutic decompression. Histology most often showed nonspecific chronic inflammation (11 patients (73.3%)). Specific histopathological diagnoses were obtained in 3 of 15 patients (20.0%): tuberculous pericarditis in one patient, tuberculous constrictive pericarditis in one patient, and metastatic adenocarcinoma in one patient. The remaining specimens showed nonspecific chronic inflammation or no specific pathological process. Median hospital stay was seven days (IQR, 6.5-8.5; range, 4-11). Two patients (13.3%) died during hospitalization; both had advanced systemic illness and preoperative tamponade physiology, and no death was attributed to a documented procedural complication.

Conclusions

Surgical drainage provided effective decompression and contributed to etiologic evaluation in selected patients with non-cardiac pericardial effusion. While specific histopathological diagnoses were uncommon, tissue sampling identified clinically important cases of tuberculosis and malignancy and provided a meaningful diagnostic contribution.

## Introduction

Pericardial effusion is the accumulation of fluid within the pericardial sac, and its causes are broad: infection, inflammation, malignancy, renal failure, autoimmune disease, trauma, and idiopathic forms all contribute [1,2]. Clinically, it spans a wide range, from a small incidental finding to a tense collection that restricts ventricular filling and produces cardiac tamponade, a true emergency [1,3]. Current guidance ties management to four factors: hemodynamic status, effusion size, inflammatory activity, and the suspected cause [1,2].

Echocardiography is the first test in suspected pericardial disease, while computed tomography and cardiac magnetic resonance add anatomical and tissue detail when the picture is unclear [1]. Where tuberculosis is common, it remains a leading cause of large pericardial effusions and constrictive pericarditis [4]. Malignancy, autoimmune disease, renal failure, and systemic illness deserve equal attention, especially when an effusion recurs, appears hemorrhagic or loculated, or is accompanied by pleural disease.

The relative frequency of these etiologies varies substantially by geographic region and local disease burden. Indonesia carries the world's second-highest tuberculosis burden after India, with roughly one million new cases each year [5]. In tuberculosis-endemic regions, tuberculosis accounts for a large share of pericardial disease, on the order of 70% of large pericardial effusions and most cases of constrictive pericarditis, compared with about 4% of pericardial effusions in industrialized countries [6]. Tuberculous pericarditis also carries substantial mortality and an appreciable risk of progression to constriction, which makes early, etiology-oriented evaluation particularly important in this setting [6].

Pericardiocentesis is usually the first-line intervention for urgent decompression of symptomatic pericardial effusion and cardiac tamponade. Current ESC guidelines recommend that management be guided by hemodynamic status, effusion characteristics, and the suspected underlying etiology [1]. While many cardiac-related effusions are managed primarily through treatment of the underlying cardiac disorder, non-cardiac etiologies such as tuberculosis, malignancy, autoimmune disease, and systemic inflammatory conditions often require additional etiologic investigation and tissue diagnosis. In these situations, surgical drainage may offer advantages beyond decompression alone by allowing direct inspection of the pericardium, acquisition of tissue for histopathological examination, and creation of a durable drainage window when recurrence is a concern. Pericardiectomy should be distinguished from drainage procedures because it is primarily reserved for constrictive pericardial disease. One caveat colors the whole field: judged by biopsy alone, the yield of a pericardial window is modest, and most specimens return as nonspecific inflammation [7-9]. That fact frames the question we set out to examine, namely, how the value of surgery should actually be measured here.

We report our experience with surgically treated pericardial effusion in a tuberculosis-endemic setting, examining echocardiographic features, etiologic patterns, surgical approaches, histopathological yield, and the broader diagnostic contribution and clinical impact of surgery in patients with non-cardiac pericardial effusion.

## Materials and methods

Study design and setting

This was a single-center, retrospective case series from a tertiary referral hospital in West Java, Indonesia. We included consecutive patients who had surgery for pericardial effusion between January 2023 and December 2025, and we report the series in line with the Preferred Reporting Of CasE Series in Surgery (PROCESS) 2020 guideline.

Patient selection

We included all consecutive patients who met the eligibility criteria during the study period. Patients qualified if echocardiography showed a moderate-to-large pericardial effusion; they subsequently underwent a surgical procedure involving pericardial drainage or resection, and the effusion was attributed to a non-cardiac etiology. Pericardial effusions were graded echocardiographically according to the maximal end-diastolic echo-free space as small (<10 mm), moderate (10-20 mm), or large (>20 mm), consistent with established guideline-based criteria. For the purposes of this study, non-cardiac etiologies were defined as pericardial effusions arising from systemic, infectious, inflammatory, malignant, renal, autoimmune, or other extracardiac conditions rather than from primary structural heart disease, primary cardiac pathology, or cardiac surgical causes. We excluded patients managed by pericardiocentesis alone, those with purely traumatic hemopericardium, and those whose operative or etiologic records were incomplete.

Surgical approach selection

The decision to perform surgical drainage or pericardiocentesis was based on clinical judgment and institutional practice rather than a standardized protocol. Pericardiocentesis was typically used for urgent decompression in patients with hemodynamic instability and in cases where the effusion was considered suitable for percutaneous drainage. Surgical intervention was generally selected for recurrent, loculated, or posterior effusions; when associated pleural pathology required concurrent drainage or pleurodesis; when tuberculosis or malignancy was suspected and tissue sampling was expected to provide diagnostic value; or when pericardiocentesis was contraindicated, unsuccessful, or followed by recurrence of the effusion. Pericardiectomy was reserved for patients with constrictive physiology supported by clinical, imaging, and intraoperative findings. Management decisions were made collaboratively by the cardiology and cardiothoracic surgery teams. Among surgically treated patients, the subxiphoid approach was usually preferred for unstable patients requiring rapid anterior decompression and drain placement, whereas video-assisted thoracoscopic surgery (VATS) was favored when pleural intervention was needed, a larger pericardial window was desired, or more representative tissue sampling was required in suspected tuberculosis or malignancy.

Data collection

Data were drawn from operative notes and medical records: age, sex, presenting symptoms, principal and supporting diagnoses, echocardiographic findings (effusion size and distribution, tamponade physiology, ventricular function), the operation performed, microbiology (culture, Gram stain, and acid-fast bacilli smear), pericardial fluid cytology, histopathology, length of stay, and in-hospital outcome. Histopathological analysis was performed on pericardial tissue specimens obtained during pericardiotomy or pericardiectomy. No myocardial or endocardial biopsies were performed. For consistency, we reported the operations as subxiphoid pericardiotomy, VATS pericardiotomy, or VATS pericardiectomy and we standardized the mixed Indonesian-English terms in the source records (for example, “subxyphoid” and “perikardiotomi”) to accepted English usage. Overt cardiac tamponade was diagnosed when chamber collapse and a swinging heart were associated with clinical hemodynamic compromise, whereas impending tamponade was defined by echocardiographic right atrial or right ventricular diastolic collapse without established hemodynamic instability. All patients underwent clinical assessment and transthoracic echocardiography before intervention. Additional imaging studies and laboratory investigations were performed as clinically indicated. Final etiologic classification was based on integrated interpretation of clinical findings, imaging studies, laboratory investigations, microbiological results, cytology, and histopathology when available.

Definitions

We defined diagnostic contribution as any information obtained through surgical drainage or tissue sampling - including histopathologic, cytologic, microbiologic, or clinically integrated findings - that helped classify the underlying etiology. This concept was broader than specific histopathological yield, which referred only to tissue-confirmed diagnoses that independently established a specific cause. We defined impact on management as any surgical finding that initiated, confirmed, or altered subsequent treatment. In-hospital mortality was death during the index admission, and procedure-related mortality was death caused directly by a technical complication of the drainage or resection.

Assessment of effusion resolution

We judged resolution by drain output rather than by repeat echocardiography. Once drainage fell to minimal or none, we considered the effusion resolved. A second in-admission echocardiogram was generally not feasible under the national insurance case-based payment scheme (Badan Penyelenggara Jaminan Sosial; INA-CBG), which represents a limitation of the study.

Statistical analysis 

Analyses were descriptive. Continuous variables are given as medians with interquartile ranges (IQRs) and ranges, and categorical variables as counts and percentages. We performed no inferential testing, given the small size and descriptive nature of the series.

Ethical considerations

The study used de-identified retrospective data and was approved by the institutional ethics committee (reference 000.9.2/297/KEPK/III/2026). Because the design was retrospective and de-identified, the committee waived individual informed consent, including for the single minor in the cohort. No identifying patient information is reported.

## Results

Fifteen patients met our criteria. Median age was 45 years (IQR, 32-55; range, 15-83), and nine (60%) were women (Table 1). All were short of breath at presentation, and dyspnea dominated the clinical picture. These were sick patients: comorbidities included end-stage renal or chronic kidney disease, systemic lupus erythematosus, thalassemia, human immunodeficiency virus (HIV) infection with pulmonary tuberculosis and breast cancer, and 10 had documented hyponatremia.

**Table 1:** Baseline patient characteristics (N = 15). IQR, interquartile range

Characteristic	Value
Age, years, median (IQR; range)	45 (32-55; 15-83)
Female sex, n (%)	9 (60.0)
Male sex, n (%)	6 (40.0)
Dyspnea at presentation, n (%)	15 (100)
Cardiac or impending tamponade, n (%)	10 (66.7)
Impending tamponade, n (%)	3 (20.0)
Overt tamponade, n (%)	7 (46.7)
Constrictive physiology, n (%)	1 (6.7)
Maximum effusion diameter, mm, range	16-50
Documented hyponatremia, n (%)	10 (66.7)
Length of stay, days, median (IQR; range)	7 (6.5-8.5; 4-11)
In-hospital mortality, n (%)	2 (13.3)

**Figure 1:**
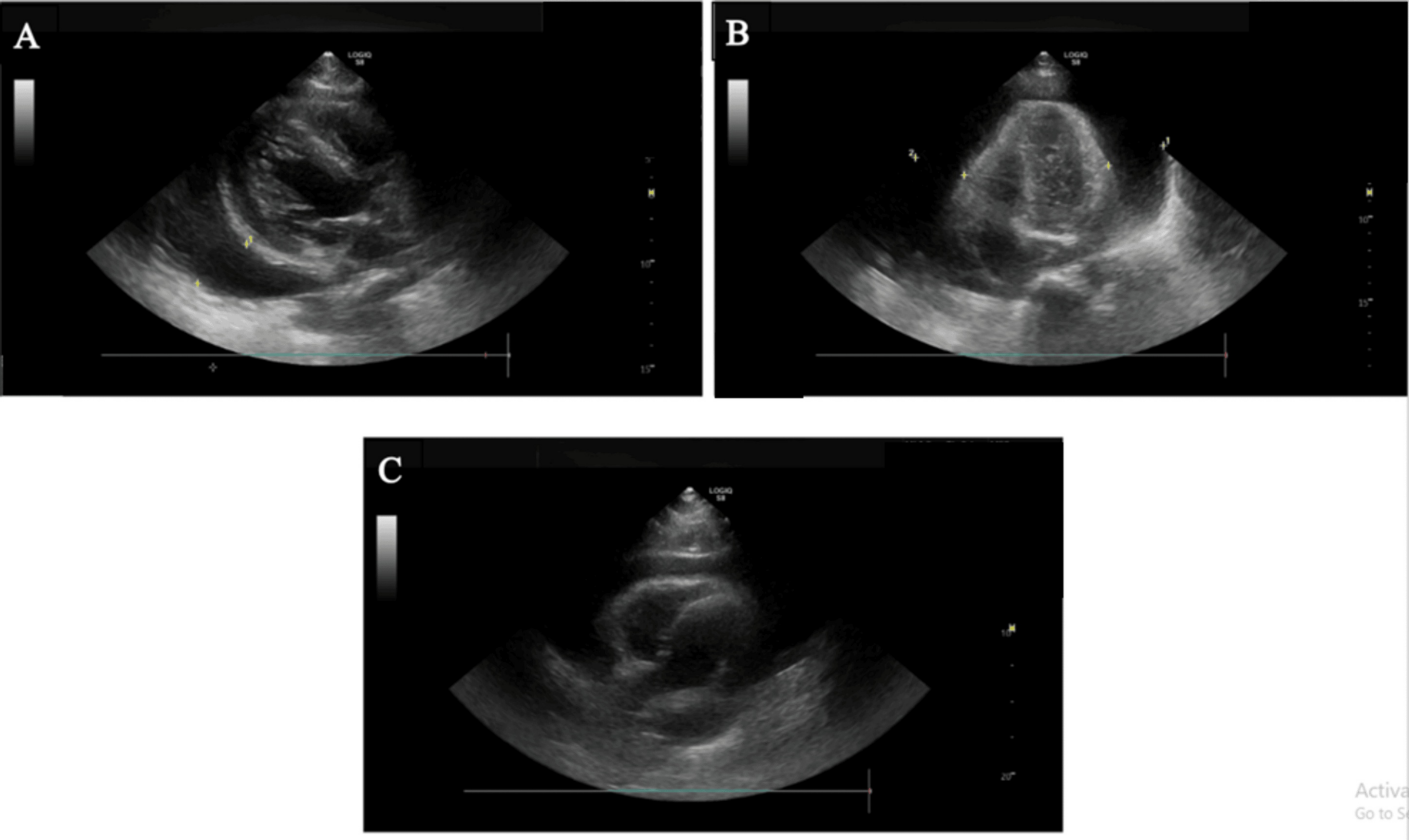
Representative transthoracic echocardiograms showing pericardial effusion in patients with non-cardiac effusion. (A, B) Moderate-to-large effusion with measured echo-free spaces; (C) large effusion surrounding the heart.

Echocardiography demonstrated a maximum effusion dimension ranging from 16 to 50 mm. Ten patients (66.7%) had tamponade physiology. Of these, seven patients (46.7%) had overt cardiac tamponade, characterized by echocardiographic chamber collapse associated with clinical hemodynamic compromise and, in several cases, a swinging heart (Figure 1). Three additional patients (20.0%) had impending tamponade, defined by right atrial or right ventricular diastolic collapse without established hemodynamic instability. One further patient had constrictive physiology, with a thickened and calcified pericardium, a septal bounce, and right atrial collapse.

**Figure 2:**
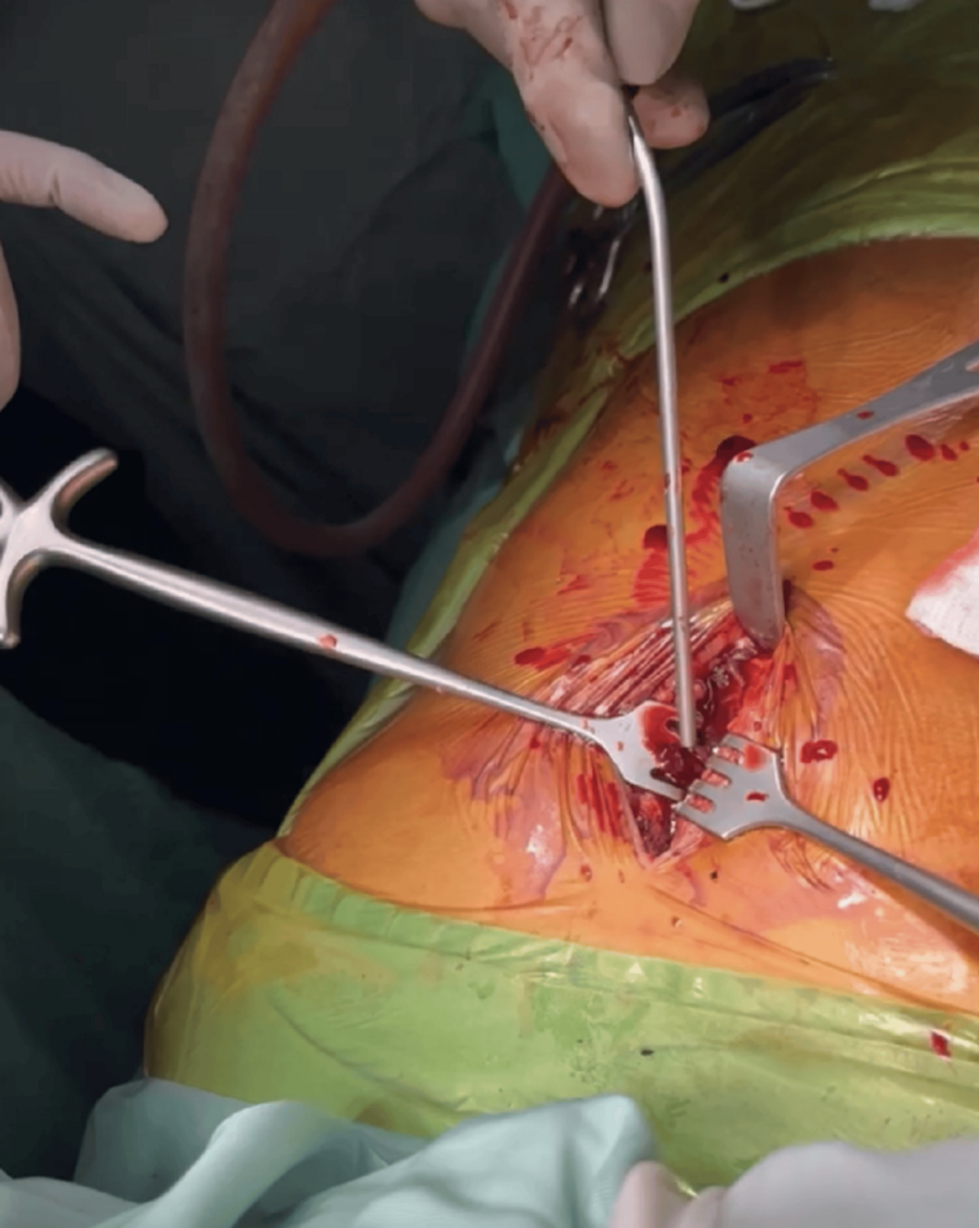
Intraoperative photograph of subxiphoid pericardiotomy. The pericardium has been opened, allowing controlled drainage of the effusion and pericardial tissue sampling.

**Figure 3:**
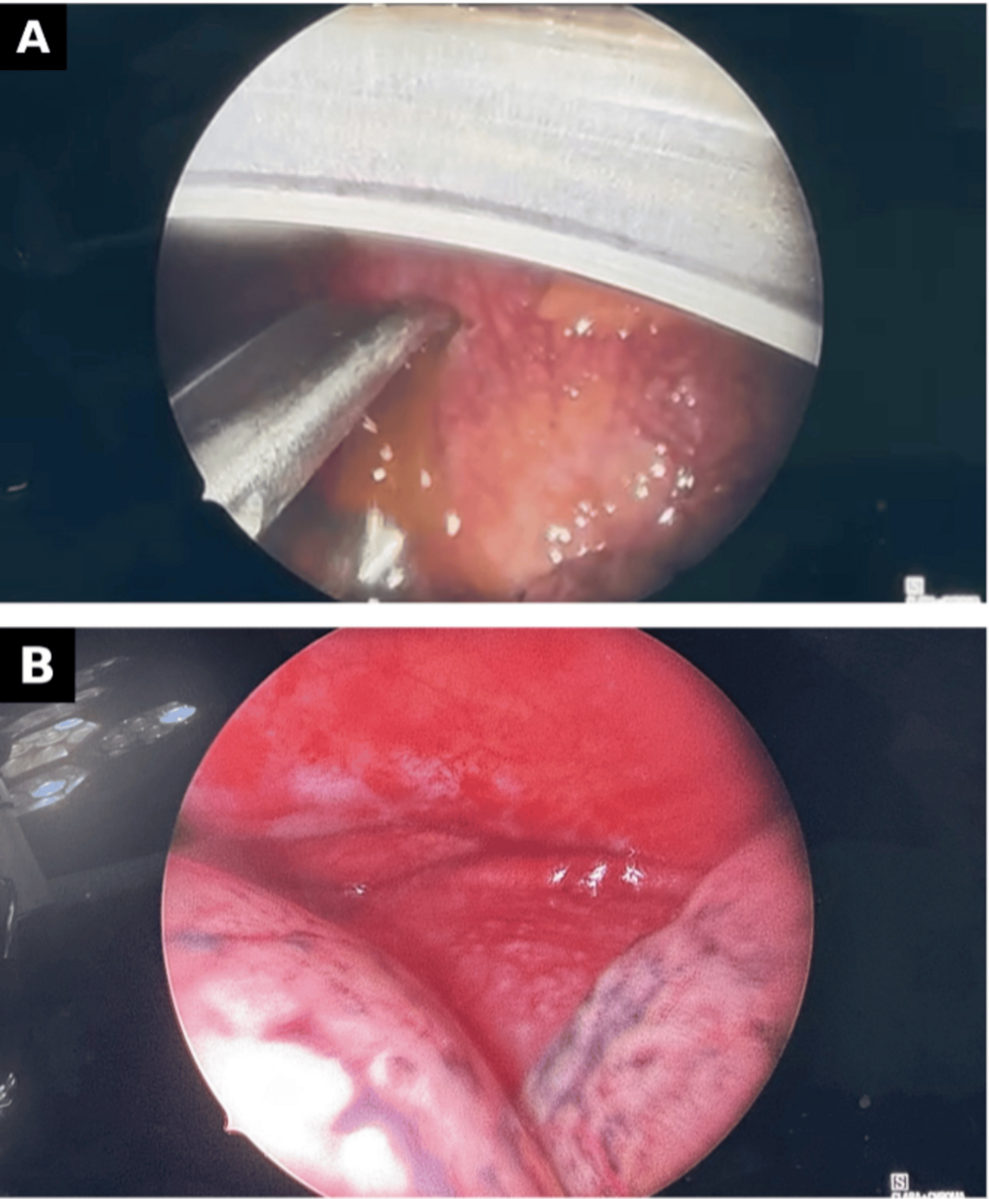
Thoracoscopic (video-assisted thoracoscopic surgery) views during pericardiectomy for tuberculous constrictive pericarditis. (A, B) The thickened pericardial surface is mobilized and resected.

Six patients (40%) had a subxiphoid pericardiotomy (Figure 2), eight (53.3%) a VATS pericardiotomy, and one (6.7%) a VATS pericardiectomy (Table 2). A drainage window was therefore the operation in 14 of 15 (93.3%), often with an added chest tube or pleurodesis, while the single pericardiectomy was done for constriction (Figure 3). We recorded no intraoperative conversion and no reoperation.

**Table 2:** Surgical approach and early outcomes. POD, postoperative day; VATS, video-assisted thoracoscopic surgery

Surgical approach / outcome	n (%)	Comment
Subxiphoid pericardiotomy	6 (40.0)	Window/drainage; chest-tube drainage in selected cases
VATS pericardiotomy	8 (53.3)	Pleural drainage or pleurodesis when indicated
VATS pericardiectomy	1 (6.7)	Performed for constrictive physiology
Total window (pericardiotomy)	14 (93.3)	Drainage rather than extensive resection
Intraoperative conversion / reoperation	0 (0)	None documented
In-hospital mortality	2 (13.3)	Early postoperative (POD 2 and POD 4); advanced systemic illness

Cultures were either sterile or grew likely skin or environmental organisms, such as coagulase-negative staphylococci, and acid-fast smears were negative in every specimen tested. Fluid cytology was usually nonspecific; it showed malignant cells (metastatic adenocarcinoma) in one patient, reactive mesothelial cells in another, and atypical cells of uncertain meaning in two more. Where biochemistry was available, the fluid was exudative, with a positive Rivalta test and a raised lactate dehydrogenase.

Histology most often showed nonspecific chronic inflammation (11 patients, 73.3%). A specific tissue diagnosis came back in three (20%): tuberculous disease in two - one of them with tuberculous constriction - and metastatic adenocarcinoma in one. In one patient (6.7%), no specific process or malignancy was found (Table 3). A detailed patient-level summary is presented in Table 4.

**Table 3:** Etiologic classification, diagnostic basis, and management impact. Specific tissue or fluid diagnosis directing targeted therapy obtained in 3 of 15 patients (20.0%). HIV, human immunodeficiency virus; TB, tuberculosis

Etiologic category	n (%)	Diagnostic basis and management impact
Tuberculous / TB-related	2 (13.3)	Tissue-confirmed tuberculous pericarditis (n=1) and tuberculous constrictive pericarditis (n=1); both established on histopathology and supported anti-tuberculous therapy.
Nonspecific inflammatory	6 (40.0)	Nonspecific chronic pericarditis on tissue and fluid; managed supportively with etiology-directed care. In one patient, *Staphylococcus* sp grew on culture, of uncertain significance.
Autoimmune-related	3 (20.0)	Systemic lupus erythematosus; nonspecific or negative tissue findings; managed medically/immunosuppression.
Hypoalbuminemia / HIV / systemic	2 (13.3)	HIV/AIDS with pulmonary TB (n=1) and thalassemia major with hypoalbuminemia (n=1); systemic disease management.
Renal-related fluid overload	1 (6.7)	End-stage renal disease with fluid overload; nonspecific pericarditis; volume/dialysis management.
Malignancy-related	1 (6.7)	Metastatic adenocarcinoma on pericardial fluid cytology and tissue; oncologic/palliative pathway.

**Table 4:** Individual patient-level summary (de-identified). Tamponade reflects echocardiographic findings; resolution after drainage was assessed by cessation of drain output (see Methods). ATT, anti-tuberculous therapy; DD, differential diagnosis; ESRD, end-stage renal disease; HIV/AIDS, human immunodeficiency virus/acquired immunodeficiency syndrome; LA, left atrium; LOS, length of stay; POD, postoperative day; RA, right atrium; RV, right ventricle; SLE, systemic lupus erythematosus; TB, tuberculosis; VATS, video-assisted thoracoscopic surgery

Pt	Age/Sex	Effusion (echo)	Tamponade	Procedure	Pericardial cytology	Histopathology	Etiologic category	LOS (d)	Outcome
1	31/M	Moderate, 18-38 mm	No	Subxiphoid pericardiotomy + chest tube	No malignant cells	Chronic nonspecific pericarditis	Renal (ESRD, fluid overload)	7	Alive
2	33/F	Massive, 20-45 mm	Yes (RA+RV collapse)	Subxiphoid pericardiotomy + chest tube	Nonspecific inflammation	Tuberculous pericarditis	Tuberculous	8	Alive
3	45/F	Severe, 20-40 mm	No	VATS pericardiotomy + chest tube	Nonspecific inflammation	Nonspecific pericarditis	Autoimmune (SLE, lupus nephritis)	10	Alive
4	55/M	Severe, 20-30 mm	Yes (swinging heart)	Subxiphoid pericardiotomy	No malignant cells	Chronic nonspecific pericarditis	HIV/AIDS, hypoalbuminemia (pulmonary TB)	8	Alive
5	54/F	Moderate-severe, 35-40 mm	Yes (swinging heart)	VATS pericardiotomy + pleurodesis	Metastatic adenocarcinoma	Metastatic adenocarcinoma	Malignancy (breast)	9	Alive
6	21/M	Mild-moderate, 26 mm	No	Subxiphoid pericardiotomy	Nonspecific inflammation	Chronic nonspecific pericarditis	Systemic (thalassemia major, hypoalbuminemia)	11	Alive
7	15/F	Severe, 26-38 mm	Yes (swinging heart)	VATS pericardiotomy	Nonspecific inflammation	Nonspecific inflammation	Autoimmune (SLE); peritoneal TB on ATT	7	Alive (transferred)
8	72/F	Large; RA collapse	Yes (RA collapse)	VATS pericardiotomy (+ lung biopsy)	Nonspecific inflammation	Chronic nonspecific pericarditis; lung: no malignancy	Nonspecific	4	Died (POD 4)
9	45/F	Large; RA collapse	Yes (RA collapse)	VATS pericardiotomy	Reactive mesothelial cells	Nonspecific pericarditis	Nonspecific (hypoalbuminemia)	5	Died (POD 2)
10	55/F	Moderate-severe, 16-25 mm	No	VATS pericardiotomy	Atypical cells, suspect metastatic carcinoma (DD reactive)	Chronic nonspecific pericarditis	Nonspecific (breast ca, post-chemo)	7	Alive
11	35/M	Severe, 30-40 mm	Yes (swinging heart)	Subxiphoid pericardiotomy	Exudate; nonspecific inflammation	Chronic nonspecific pericarditis	Nonspecific (cervical lymphadenopathy)	6	Alive
12	44/F	Moderate-severe, 25-30 mm	Yes (swinging heart)	Subxiphoid pericardiotomy	Nonspecific inflammation	Chronic nonspecific pericarditis	Nonspecific	7	Alive
13	83/M	Severe, ~50 mm	Yes (swinging heart)	VATS pericardiotomy	Nonspecific inflammation	Chronic nonspecific pericarditis	Nonspecific, non-TB (*Staphylococcus* sp)	7	Alive
14	60/M	Constrictive; thickening + calcification	Impending (septal bounce, RA collapse)	VATS pericardiectomy	Lymphocytic effusion, suspected TB	Constrictive pericarditis due to TB	Tuberculous	6	Alive
15	19/F	Severe, 40-50 mm	No	VATS pericardiotomy	No malignant cells	No specific process or malignancy	Autoimmune (SLE), hypoalbuminemia	9	Alive

Surgical drainage was successfully completed in all 15 patients and provided therapeutic decompression. In addition, tissue and fluid samples were obtained for diagnostic evaluation. A specific diagnosis was established in three patients (20%): tuberculous pericarditis and tuberculous constrictive pericarditis, which justified anti-tuberculous therapy (and, in the second, relief of constriction by pericardiectomy), and metastatic adenocarcinoma, which confirmed malignant involvement and pointed toward oncologic and palliative care. In the remaining patients, sampling ruled out malignancy and granulomatous disease and, read alongside the clinical, microbiologic, and imaging data, supported an etiologic label and a plan. It is worth noting that the atypical cytology seen in two patients was not borne out as malignancy on tissue.

Median stay was seven days (IQR, 6.5-8.5; range, 4-11). Two patients (13.3%) died during hospitalization; both had advanced systemic illness and preoperative tamponade physiology, and no death was attributed to a documented procedural complication.

## Discussion

In this series, the pericardial effusions that reached the operating room were overwhelmingly non-cardiac in origin. Nonspecific inflammatory pericarditis formed the largest single group, accompanied by tuberculous and TB-related disease, autoimmune effusion, systemic illness, renal fluid overload, and malignancy. Geography explains much of this: where tuberculosis is endemic, it stays near the top of the differential for any large effusion or new constriction [4]. The heavy comorbidity load we saw, including HIV with pulmonary tuberculosis, lupus, thalassemia, and malignancy, is a reminder that these effusions rarely stand alone.

When to operate comes down to hemodynamics, the chance of recurrence, the suspected cause, and how much a tissue diagnosis is likely to help. Guidelines support urgent drainage for tamponade and for symptomatic moderate-to-large effusions, with cause-directed therapy carrying the longer-term work [1,2]. Here, surgery relieved the effusion in every patient and let us sample the pericardium. That VATS and the subxiphoid route carried the series fits the literature, which treats both as standard drainage options [10,11]. The choice between subxiphoid and thoracoscopic (VATS) pericardiotomy was based on clinical presentation and associated thoracic pathology rather than a strict predefined protocol. Subxiphoid pericardiotomy was performed through a small subxiphoid incision to create a pericardial window, drain the effusion, obtain tissue samples, and place a drainage catheter. We generally preferred this approach in hemodynamically unstable patients or those with cardiac tamponade requiring prompt decompression through a direct anterior approach. VATS pericardiotomy was performed thoracoscopically under general anesthesia, allowing creation of a wider pericardial fenestration with concomitant pleural inspection, drainage, biopsy, or pleurodesis when indicated. Accordingly, VATS was favored in hemodynamically stable patients when concomitant pleural disease required evaluation or treatment, when pleurodesis was planned, or when a wider pericardial fenestration was desired. Surgeon judgment and expertise also contributed to procedural selection.

The heart of this study is the gap between biopsy yield and diagnostic value. Older surgical series make the point. Volk et al. found diagnostic information in only 17.2% of 145 patients drained surgically, and biopsy added little to cytology [7]. Giuliani et al. reported that most non-targeted pericardial biopsies were nonspecific, with a firm histologic diagnosis in the minority [8]. Yildirim et al. described the same pattern from a single center: combined fluid and biopsy analysis helped, but selectively [9].

Our numbers sit squarely in that tradition. Specific histology turned up in three patients (20%), namely tuberculous pericarditis, tuberculous constriction, and metastatic adenocarcinoma, while the rest were nonspecific. We would not want these data read as a case for biopsying every pericardium in the hope of a high yield. The contribution of surgery should be interpreted differently. It combined decompression, fluid study, and tissue with the rest of the clinical information and, measured as impact on management, directly informed or supported subsequent treatment decisions in those three patients. In the others, it excluded malignancy or granulomatous disease and sharpened the working diagnosis. These negative findings helped support the management of other likely causes and avoided unnecessary treatment for tuberculosis or malignancy. In selected non-cardiac effusions, then, the honest description of surgery’s role is a diagnostic contribution read in context, not a yield figure. In our series, tissue sampling was most useful in patients with suspected tuberculosis or malignancy, such as those with pleural disease or known or suspected cancer.

The difference between the two operations matters just as much. A pericardiotomy opens a window to drain fluid and take a sample; a pericardiectomy removes the pericardium to relieve constriction or clear diseased tissue. In our group, 14 of 15 procedures were windows, and we reserved pericardiectomy for the one patient with tuberculous constriction, whose pericardium was thick and calcified with a septal bounce and right atrial collapse. That matches the view that pericardiectomy belongs to chronic or irreversible constriction and to selected refractory cases [12]. However, because only one patient underwent pericardiectomy in this series, no meaningful conclusions can be drawn regarding the efficacy, safety, or outcomes of pericardiectomy. This case should therefore be interpreted as illustrative of the procedure’s role in selected patients rather than as evidence regarding its efficacy or safety. One caveat is in order about the thoracoscopic resection we performed: it was a limited operation matched to that patient's physiology. For established, calcified, or circumferential constriction, a fuller pericardiectomy through sternotomy or thoracotomy is usually needed, and how adequate a thoracoscopic resection is in those settings is a fair question.

Two patients (13.3%) died during the index admission, on postoperative days 2 and 4. Outcome assessment was limited to the index hospitalization. No documented intraoperative conversion, repeat surgical intervention, or major procedure-related complication was recorded during the admission. The two early postoperative deaths occurred in patients with advanced systemic illness and preoperative tamponade physiology. Although neither death was attributed to a documented procedural complication and no technical complication of the drainage procedure was identified, the retrospective design limits definitive determination of the immediate cause of death. Therefore, these outcomes should be interpreted in the context of severe underlying disease rather than as evidence of procedure-related mortality.

Practically, early surgical referral makes sense when an effusion is recurrent, loculated, or posterior; when it is associated with pleural disease; when tuberculosis or malignancy is suspected and tissue diagnosis is likely to influence treatment; or when pericardiocentesis has failed or is considered unsafe. The reverse is also true. Pericardiocentesis remains appropriate for rapid decompression in many unstable patients and for idiopathic or presumed viral effusions when tissue diagnosis is unlikely to alter management [1,2,13].

The limitations are real. This was a small, retrospective, single-center study without a comparison group treated by pericardiocentesis alone, limiting assessment of the relative diagnostic value of surgery. Effusion resolution was assessed by drain output rather than repeat echocardiography, so residual or recurrent effusions may have been missed. Tuberculosis was diagnosed from histology and clinical findings without Xpert MTB/RIF or adenosine deaminase testing, which may have led to underdiagnosis. Etiologic classification was based on available records; many specimens were nonspecific, and the exact causes of death could not always be determined reliably. Follow-up beyond hospital discharge was limited, preventing reliable assessment of recurrence, late complications, reintervention, and long-term outcomes. Even so, the series reflects real-world surgical management of pericardial effusion in a tuberculosis-endemic setting.

## Conclusions

Surgical management of pericardial effusion can relieve pressure and help determine the cause in selected patients with non-cardiac conditions. Although many tissue samples show nonspecific results, some cases reveal clear diagnoses such as tuberculosis or cancer, which guide subsequent management decisions. The benefit of surgery is best understood when combined with clinical, laboratory, and imaging findings, not from tissue results alone. Despite limitations such as small sample size and retrospective design, these findings support a selective approach to surgery, especially when tuberculosis or malignancy is suspected and a tissue diagnosis may affect treatment decisions.
